# Estrogen receptor beta signaling enhances extinction memory recall for heroin-conditioned cues in a sex- and region-specific manner

**DOI:** 10.1038/s41398-024-03001-y

**Published:** 2024-07-12

**Authors:** Jordan S. Carter, Caitlyn C. Costa, Stacia I. Lewandowski, Katharine H. Nelson, Sarah T. Goldsmith, Michael D. Scofield, Carmela M. Reichel

**Affiliations:** Department of Neuroscience, Medical University of South Carolina, Charleston, SC 29425 USA

**Keywords:** Learning and memory, Addiction

## Abstract

Return to use, or relapse, is a major challenge in the treatment of opioid use disorder (OUD). Relapse can be precipitated by several factors, including exposure to drug-conditioned cues. Identifying successful treatments to mitigate cue-induced relapse has been challenging, perhaps due to extinction memory recall (EMR) deficits. Previously, inhibition of estradiol (E2) signaling in the basolateral amygdala (BLA) impaired heroin-cue EMR. This effect was recapitulated by antagonism of BLA estrogen receptors (ER) in a sex-specific manner such that blocking ERα in males, but ERβ in females, impaired EMR. However, it is unclear whether increased E2 signaling, in the BLA or systemically, enhances heroin-cue EMR. We hypothesized that ERβ agonism would enhance heroin-cue EMR in a sex- and region-specific manner. To determine the capacity of E2 signaling to improve EMR, we pharmacologically manipulated ERβ across several translationally designed experiments. First, male and female rats acquired heroin or sucrose self-administration. Next, during a cued extinction session, we administered diarylpropionitrile (DPN, an ERβ agonist) and tested anxiety-like behavior on an open field. Subsequently, we assessed EMR in a cue-induced reinstatement test and, finally, measured ERβ expression in several brain regions. Across all experiments, females took more heroin and sucrose than males and had greater responses during heroin-cued extinction. Administration of DPN in the BLA enhanced EMR in females only, driven by ERβ’s impacts on memory consolidation. Interestingly, however, systemic DPN administration improved EMR for heroin cues in both sexes across several different tests, but did not impact sucrose-cue EMR. Immunohistochemical analysis of ERβ expression across several different brain regions showed that females only had greater expression of ERβ in the basal nucleus of the BLA. Here, in several preclinical experiments, we demonstrated that ERβ agonism enhances heroin-cue EMR and has potential utility in combatting cue-induced relapse.

## Introduction

Rates of opioid use disorder (OUD) remain at epidemic levels, contributing to increased opioid-related overdoses and a substantial annual economic burden [1–3]. Return to use, or relapse, is the major challenge in treating OUD. Several factors can contribute to relapse, including psychological distress, withdrawal, craving, re-exposure to the drug, and/or drug-conditioned environmental cues [4–8]. Currently, several behavioral and pharmacologic treatments, often administered in combination, help manage most of these factors [9]. However, only one behavioral intervention has been leveraged to target cue reactivity in substance use disorders (SUDs): cue exposure therapy (CET) [10, 11]. CET aims to prevent cue-induced relapse by presenting drug-related cues to extinguish cue reactivity and enhance extinction memory formation [11]. Despite mixed results in treatment of SUDs with CET [11–15], interest in pharmacologic adjuncts to improve CET’s therapeutic benefit remains strong [16, 17]. In particular, the only study of CET for the treatment of OUD found increased dropout and relapse rates, despite decreases in physiological cue reactivity [12]. By addressing cue reactivity, CET could be especially advantageous for treating women with OUD. Women report greater cue-induced opioid craving than men [18], have lower treatment utilization [19–21], and are bearing an increasing burden of the OUD epidemic [22].

During chronic opioid use, discrete sensory cues in the environment become conditioned with the reinforcing effects of the drug. Exposure to these cues during abstinence can increase drug craving, leading to relapse [7, 23, 24]. Drug-conditioned cues maintain their power to motivate drug-seeking even after repeated unreinforced cue exposures (extinction-resistant) [24–26] and can evoke even greater drug-seeking behavior as abstinence is prolonged (incubation of drug craving) [27, 28]. CET does decrease physiological cue reactivity in individuals with OUD, indicating that they are able to extinguish the drug-cue association, but participants did not report changes in subjective cue reactivity or craving [12]. Thus, a key pathologic feature in individuals with OUD may be a deficit in extinction memory recall (EMR), whereby opioids induce a durable drug-conditioned memory (pro-relapse) that overrides the poorly retained extinction memory (pro-abstinence) when the individual is exposed to drug-associated cues that exacerbate craving [4, 29]. Dysfunction of the basolateral amygdala complex (BLA) could underlie these deficits, as the BLA is extensively involved in formation, extinction, and recall of conditioned and extinguished associations for rewarding stimuli [30].

Estradiol (E2), the major endogenous form of estrogen, is one candidate for improving CET’s efficacy. In the brain, E2 is a neurosteroid with functional roles in memory and synaptic plasticity [31–33], as well as influences on opioid receptor signaling [34]. While E2 from the peripheral circulation can enter the central nervous system, the major source of central E2 in both males and females is from local de novo synthesis or aromatization of testosterone [32, 33, 35, 36]. Importantly, males and females express similar levels of aromatase (the final enzyme in E2 synthesis) on several different cell types and produce similar levels of E2 in the BLA, so contributions of E2 signaling must be studied in both sexes [37]. Following synthesis, E2 binds to estrogen receptors (ERs; ERα, ERβ, and GPER) which have diverse signaling properties and mediate a wide range of effects [38]. While ER expression has been documented throughout the brain, including the BLA, prior studies of ERβ expression specifically might be unreliable due to problems with antibody specificity [39] and poor interstudy replicability [40–42].

E2’s theoretical utility as an adjunct to CET, to enhance drug-cue EMR, is based on prior studies of fear and reward EMR. In females, increased E2 signaling enhances fear EMR [43–45], an effect mediated by ERβ, not ERα [45]. While E2’s enhancing effects have not been tested in males, inhibition of E2 synthesis impairs fear EMR in males [46]. Impaired E2 signaling in females also decreases fear EMR [47]. Interestingly, while only ERβ agonism enhances fear EMR at baseline [45], both ERα and ERβ agonism rescue fear EMR when it is impaired by hormonal contraceptives [47]. Similarly, in a prior study, we found that inhibition of E2 synthesis in the BLA impairs EMR of a heroin-conditioned stimulus [48]. This study identified the BLA as a brain region in which E2 signaling impacts cue EMR, and was the first to evaluate EMR for a rewarding stimulus. Interestingly, ERβ antagonism in the BLA recapitulates this EMR impairment in females, while ERα antagonism impairs heroin-cue EMR in males [48]. To our knowledge, no studies to date have evaluated the potential for E2 signaling to enhance heroin-cue EMR.

Our primary goal herein is to test the contributions of ERβ signaling to heroin-cue EMR through a series of translationally designed experiments in male and female rats. Our decision to focus on ERβ here was driven by several factors: the relative lack of studies on ERβ, particularly in the BLA; prior studies showing that ERβ enhances fear EMR at baseline [45] and in impaired states [47]; and our finding that inhibition of ERβ signaling in the BLA impairs heroin-cue EMR in females [48], who are understudied in basic and translational research. Our overarching hypothesis is that ERβ agonism enhances heroin-cue EMR in a sex-specific manner. To test this, first, we examined heroin-cue EMR following ERβ agonism in the BLA (Exp. 1) or after systemic agonist administration (Exp. 2). Next, we tested ERβ agonism’s effects on EMR after protracted abstinence (Exp. 3), and whether these effects are maintained following post-treatment abstinence (Exp. 4). We also assessed the effects on sucrose-cue EMR (Exp. 5). Finally, to determine whether sex-specific expression of ERβ may contribute to behavioral differences, we quantified ERβ expression across several brain regions, an endeavor which has previously had numerous challenges with reliability and replicability.

## Materials and methods

A complete list of materials and detailed methods are included in the Supplement.

### Subjects and surgeries

A total of 158 male and female Wistar rats (8–9 weeks old, Envigo, Indianapolis, IN) were used. All procedures were approved by the Institutional Animal Care and Use Committee of the Medical University of South Carolina in accordance with the “Guide for the Care and Use of Laboratory Rats” of the Institute of Laboratory Animal Resources on Life Sciences, National Research Council. Following acclimation, subjects had a catheter implanted into the jugular vein for heroin self-administration (Exps. 1–4). Rats in Experiment 1 also had bilateral cannulas implanted in the BLA (relative to bregma: anterior-posterior: –2.5 mm, medial-lateral: ±4.8 mm, dorsal-ventral: –8.5 mm). Surgical details are in the Supplement and [48]. Estrous cycle was not tracked, as cycle phase does not affect heroin-cue EMR [48].

### Drugs and delivery

Heroin (NIDA Drug Supply Program, Rockville, MD) was dissolved in saline to 200 mg/250 mL. Sucrose pellets (45 mg; Bio-Serv, Flemington, NJ) were placed in hoppers. The ERβ agonist (diarylpropionitrile, DPN, MedChemExpress, Monmouth Junction, NJ) was dissolved in DMSO and diluted with saline. Rats were randomly assigned to receive DPN or vehicle. Intracranial (IC) infusions of DPN into the BLA (Exp. 1; 1 µL at 0.2 µL/min) yielded a dose of 10 pg, bilaterally. Infusion localization/spread was previously validated [48]. Subcutaneous (SC) DPN injections (Exps. 2–5) were 1.0 mg/kg. Doses were selected from published literature [49–53].

### Self-administration, cued extinction, extinction memory recall, and open field test

**Fig. 1:**
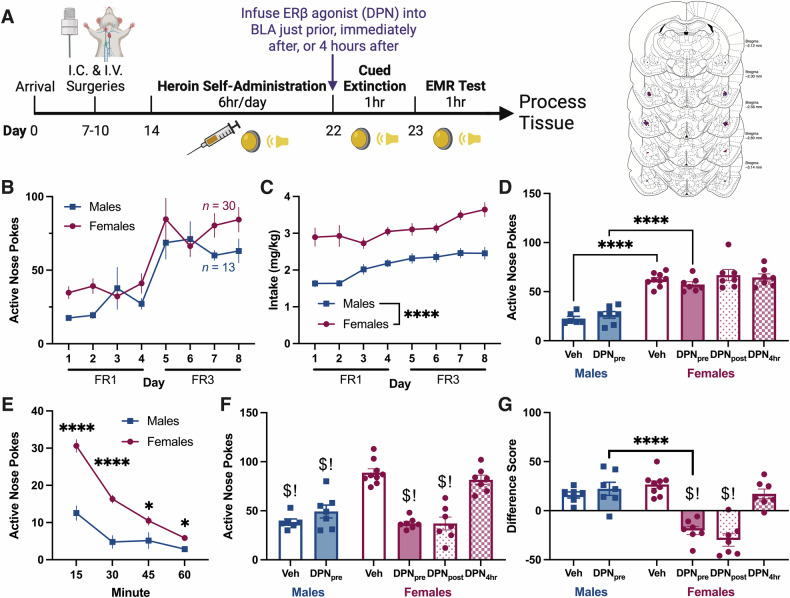
Experiment 1: ERβ agonism in the BLA during cued extinction enhances heroin-cue EMR in a sex-specific manner. **A** Experiment 1 timeline and cannula placements. **B** Daily ANPs during heroin self-administration in males and females. ANPs did not differ between the sexes. **C** Heroin intake (mg heroin/kg body weight) during self-administration. Females took more heroin than males (ME of sex). **D** Total ANPs during the 1 h cued extinction session. Treatment-matched post-hoc comparisons of males and females show that females had greater ANPs than males. However, ERβ agonism did not impact ANPs. **E** Quarterly ANPs during the cued extinction session. Veh and DPN treated groups were collapsed since DPN had no effect. At all timepoints, but particularly during the start of the session, females had greater ANPs than males (time x sex interaction). **F** Total ANPs during the 1 h EMR test. DPN_pre_ and DPN_post_ females had lower ANPs relative to Veh and DPN_4h_ females. Males ANPs did not change in response to DPN, but they were also lower than Veh and DPN_4h_ females. **G** Difference scores comparing ANPs during extinction to ANPs during test. DPN_pre_ and DPN_post_ females had decreased difference scores relative to all other groups. Difference scores in Veh and DPN_4h_ females were not different from males. All data are shown as mean ± SEM; *n* = 6–7 rats/group; *p < 0.05, ****p < 0.0001, ^$^*p* < 0.0001 versus Veh females, !*p* < 0.0002 versus DPN_4h_ females. ANPs, active nose pokes; BLA, basolateral amygdala; DPN, diarylpropionitrile (ERβ agonist); EMR, extinction memory recall; FR, fixed ratio; INPs, inactive nose pokes; ME, main effect; Veh, vehicle.

**Fig. 2:**
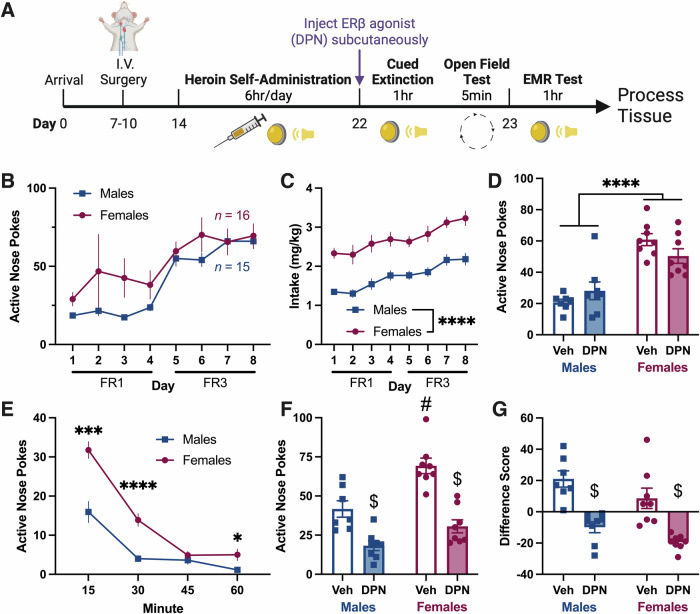
Experiment 2: Systemic ERβ agonism enhances heroin-cue EMR in both sexes. **A** Experiment 2 timeline. **B** Daily ANPs during heroin self-administration in males and females. ANPs did not differ between the sexes. **C** Heroin intake (mg heroin/kg body weight) during self-administration. Females took more heroin than males (ME of sex). **D** Total ANPs during the 1 h cued extinction session. Females had greater ANPs than males. Systemically administered DPN had no effect on ANPs during extinction in either sex. **E** Quarterly ANPs during the cued extinction session. Veh and DPN-treated groups were collapsed since DPN had no effect. Female ANPs were increased relative to males, particularly early on (time x sex interaction). **F** Total ANPs during the 1 h EMR test. ANPs were impacted by DPN (ME of DPN) and sex (ME of sex). DPN, administered systemically the day prior, decreased ANPs in both sexes relative to Veh controls. Veh females had greater ANPs than all other groups. **G** Difference scores comparing ANPs during extinction to ANPs during test. Difference scores were also impacted by DPN (ME of DPN) and sex (ME of sex). DPN decreased difference scores in both sexes relative to sex matched Veh controls. All data are shown as mean ± SEM; *n* = 7–8 rats/group; *p < 0.05, ***p < 0.001, ****p < 0.0001, ^$^*p* < 0.05 versus Veh, ^#^*p* < 0.05 versus all other groups. ANPs, active nose pokes; DPN, diarylpropionitrile (ERβ agonist); EMR, extinction memory recall; FR, fixed ratio; INPs, inactive nose pokes; ME, main effect; Veh, vehicle.

**Fig. 3:**
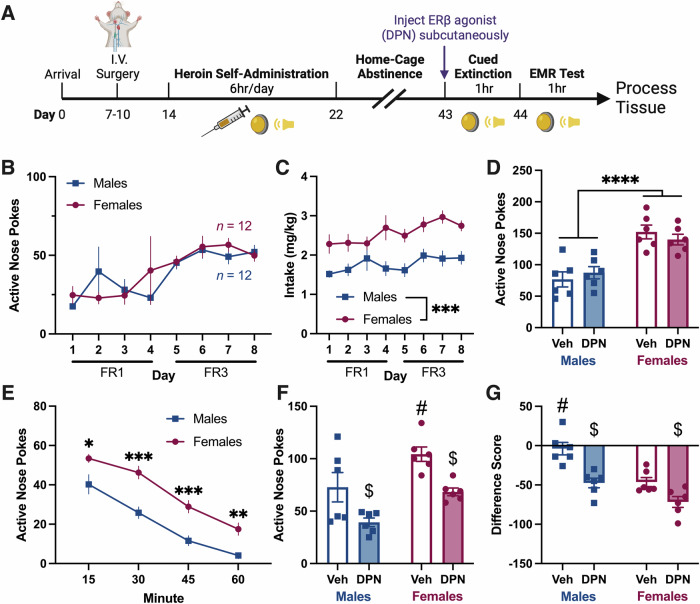
Experiment 3: Systemic ERβ agonism enhances heroin-cue EMR after abstinence. **A** Experiment 3 timeline. **B** Daily ANPs during heroin self-administration in males and females. ANPs did not differ between the sexes. **C** Heroin intake (mg heroin/kg body weight) during self-administration. Females took more heroin than males (ME of sex). **D** Total ANPs during the 1 h cued extinction session following 21 days of abstinence. Females had greater ANPs than males (ME of sex). DPN had no effect on ANPs during extinction in either sex. **E** Quarterly ANPs during the cued extinction session. Veh and DPN treated groups were collapsed since DPN had no effect. Female ANPs were increased relative to males throughout the session (ME of sex). **F** Total ANPs during the 1 h EMR test. ANPs were impacted by DPN (ME of DPN) and sex (ME of sex). DPN, administered systemically the day prior, decreased ANPs in both sexes relative to Veh controls. Veh females had greater ANPs than all other groups. **G** Difference scores comparing ANPs during extinction to ANPs during test. Difference scores were also impacted by DPN (ME of DPN) and sex (ME of sex). DPN decreased difference scores in both sexes relative to Veh controls. Veh males had greater difference scores than all other groups. All data are shown as mean ± SEM; *n* = 6 rats/group; *p < 0.05, **p < 0.01, ***p < 0.001, ****p < 0.0001, ^$^*p* < 0.05 versus Veh, ^#^*p* < 0.05 versus all other groups. ANPs, active nose pokes; DPN, diarylpropionitrile (ERβ agonist); EMR, extinction memory recall; FR, fixed ratio; INPs, inactive nose pokes; ME, main effect; Veh, vehicle.

**Fig. 4:**
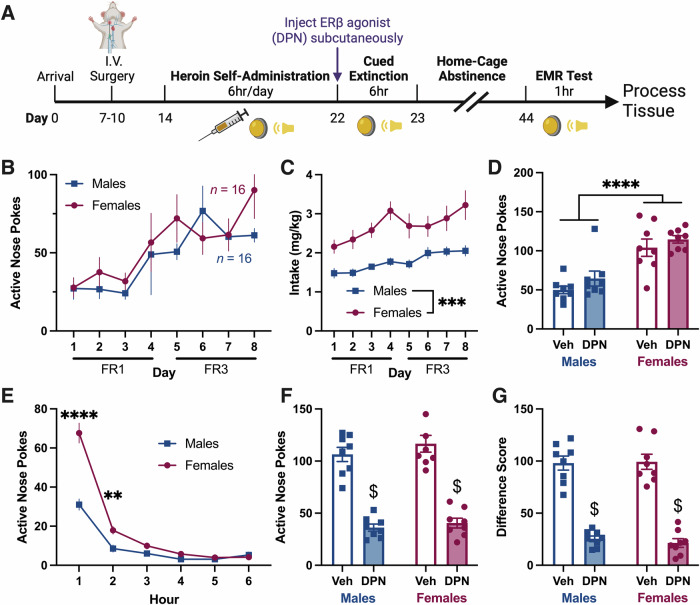
Experiment 4: Systemic ERβ agonism maintains enhancing effects on heroin-cue EMR following post-treatment abstinence*.* **A** Experiment 4 timeline. **B** Daily ANPs during heroin self-administration in males and females. ANPs did not differ between the sexes. **C** Heroin intake (mg heroin/kg body weight) during self-administration. Females took more heroin than males (ME of sex). **D** Total ANPs during the 6 h cued extinction session. Females had greater ANPs than males (ME of sex). ERβ agonism had no effect on ANPs during extinction in either sex. **E** Hourly ANPs during the cued extinction session. Veh and DPN treated groups were collapsed since DPN had no effect. Female ANPs were increased relative to males early in the session (time x sex interaction). **F** Total ANPs during the 1 h EMR test following 21 days of abstinence. ANPs were impacted by DPN only (ME of DPN). DPN, administered systemically 21 days prior, decreased ANPs in both sexes relative to Veh controls. **G** Difference scores comparing ANPs during extinction to ANPs during test. Difference scores were also only impacted by DPN (ME of DPN). DPN decreased difference scores in both sexes relative to Veh controls. All data are shown as mean ± SEM; *n* = 8 rats/group; **p < 0.01, ***p < 0.001, ****p < 0.0001, ^$^*p* < 0.05 versus Veh. ANPs, active nose pokes; DPN, diarylpropionitrile (ERβ agonist); EMR, extinction memory recall; FR, fixed ratio; INPs, inactive nose pokes; ME, main effect; Veh, vehicle.

**Fig. 5:**
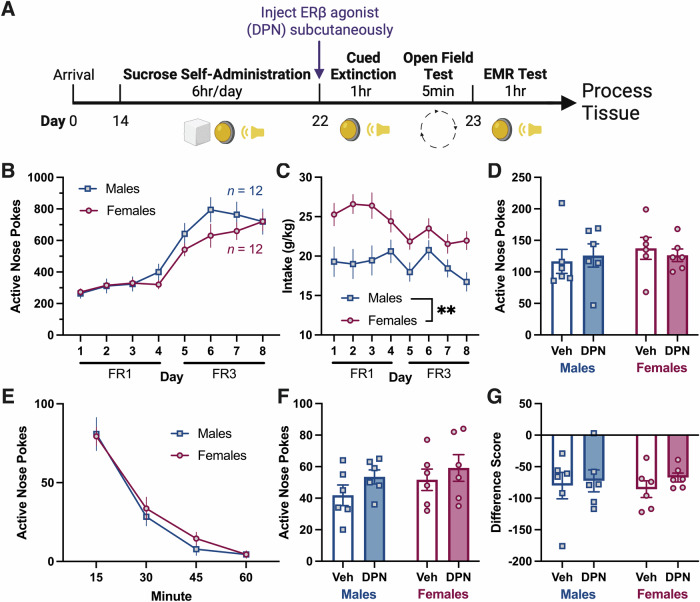
Experiment 5: Systemic ERβ agonism has no effect on sucrose-cue EMR. **A** Experiment 5 timeline. **B** Daily ANPs during sucrose self-administration in males and females. ANPs did not differ between the sexes. **C** Sucrose intake (g sucrose/kg body weight) during self-administration. Females took more sucrose than males (ME of sex). **D** Total ANPs during the 1 h cued extinction session. Females and males had similar ANP responding regardless of DPN. **E** Quarterly ANPs during the cued extinction session. Veh and DPN treated groups were collapsed since DPN had no effect. ANPs did not differ by sex during the session. **F** Total ANPs during the 1 h EMR test. DPN, administered systemically the day prior, did not impact ANPs in either sex. **G** Difference scores comparing ANPs during extinction to ANPs during test. DPN had no effect on difference scores in either sex. All data are shown as mean ± SEM; *n* = 6 rats/group; **p < 0.01. ANPs, active nose pokes; DPN, diarylpropionitrile (ERβ agonist); EMR, extinction memory recall; FR, fixed ratio; INPs, inactive nose pokes; ME, main effect; Veh, vehicle.

Behavior occurred as described in the Supplement and [48], summarized in timelines for each experiment (Figs. 1–5, panels A). Rats nose-poked to self-administer heroin (Exps. 1–4) or sucrose (Exp. 5) for 6 h/day for 8 consecutive days. Active nose pokes (ANPs) delivered a 40 µg heroin infusion or 45 mg sucrose pellet. Reward delivery was paired with a 5-sec light and tone stimulus (heroin- or sucrose-conditioned cue). Inactive nose pokes (INPs) were without consequence. The first four days were on a fixed ratio (FR) 1 reinforcement schedule, followed by four days at FR3.

After self-administration, rats underwent cued extinction, akin to CET in humans. Session duration was varied to prevent (1 h, Exps. 1–3, 5) or promote (6 h, Exp. 4) EMR in control subjects. Rats in Experiment 3 had 21 days of abstinence between self-administration and extinction, during which rats remained in their home cage but were handled daily. During cued extinction, ANPs led to presentation of the conditioned cue without drug delivery. DPN/vehicle was administered before or after cued extinction: IC (Exp. 1, 10 min prior [DPN_pre_], immediately after [DPN_post_], or 4 h after [DPN_4hr_]) or SC (Exps. 2–5, 20 min prior [DPN]). Subjects in Experiments 2 and 5 underwent an open field test after cued extinction (Supplement).

Subsequently, rats were tested for EMR (EMR Test). Subjects in Experiments 1–3 and 5 tested the day after extinction. Subjects in Experiment 4 had 21 days of home-cage abstinence between extinction and EMR test. Conditions during the 1 h test session were as in cued extinction (ANPs resulted in presentation of the light/tone cue without drug delivery), except there was no DPN/vehicle administration.

### Immunohistochemistry and confocal microscopy

Brains from Experiment 1 were checked for cannula placements. Brains from Experiments 2 and 5 were labeled for ERβ via immunohistochemistry (IHC) using a rigorously validated monoclonal antibody [39, 54] and imaged via confocal microscopy (Supplement).

### Data and statistical analysis

Sample sizes were estimated from prior experiments [48]. All subjects who completed the experiments as described were used in the subsequent statistical analyses. To control for individual differences in levels of operant responding when assessing EMR, we calculated a difference score (*ANPs*_*EMR Test*_ *[total] – ANPs*_*Extinction*_ *[average/hour]*) [48]. This allows for direct, standardized, within-subjects comparisons of responses between cued extinction and EMR test across experiments. All analyses and graphs were produced using Prism (v.10, GraphPad). Dependent measures (nose pokes, difference scores, intake, locomotor activity, ERβ expression) were analyzed using t-tests (paired/unpaired ± nesting) or analyses of variance (ANOVAs; 1 or 2way ± repeated measures [RM] and/or nesting; with Geisser-Greenhouse corrections). For all ANOVAs, the between subjects’ independent variables were sex (male/female) and treatment during extinction (Veh/DPN) with Holm-Šídák’s post-hoc comparisons as appropriate. The significance was set at α = 0.05 throughout. Complete statistics are included in Tables S1–S7. INPs during cued extinction and EMR test did not differ by sex or treatment in any Experiments (Table S8). Additional behavioral data (INPs during self-administration, heroin infusions/sucrose deliveries, and direct comparisons of ANP responding between extinction and EMR test) are shown in Figures S2 (Exp. 1) and S3 (Exps. 2–5).

## Results

### ERβ agonism in the BLA during cued extinction enhances heroin-cue EMR in a sex-specific manner

In Experiment 1, we tested whether ERβ agonism in the BLA during cued extinction would enhance heroin-cue EMR (Fig. 1A). Cannula placements are shown in Fig. 1A (enlarged, Figure S1). During heroin self-administration, ANPs were similar between both sexes (Fig. 1B), but INPs were greater in females (Figure S2A, 2way RM ANOVA, main effect of sex, [*F*_1,41_ = 4.52, *p* = 0.040]). Heroin intake (mg/kg) was also higher in females (Fig. 1C, 2way RM ANOVA, main effect of sex, [*F*_1,41_ = 36.26, *p* < 0.0001]). ANPs during cued extinction differed across groups (Fig. 1D, 1way ANOVA, [*F*_5,37_ = 31.22, *p* < 0.0001]), but post-hocs indicate these differences were driven by sex, not DPN. Comparison of ANP responding across the extinction session resulted in a significant time × sex interaction (Fig. 1E, 2way RM ANOVA, [*F*_3,123_ = 8.71, *p* < 0.0001]). Females had elevated ANPs relative to males at all timepoints across the extinction session, but particularly early on.

ERβ agonism in the BLA before extinction had sex- and timepoint-dependent effects on EMR, as assessed by ANPs during test (Fig. 1F, 1way ANOVA, [*F*_5,37_ = 25.39, *p* < 0.0001]) and difference scores (Fig. 1G, 1way ANOVA, [*F*_5,37_ = 23.24, *p* < 0.0001]). Post-hocs revealed that vehicle and DPN_4h_ females had greater ANPs during test than all other groups (Fig. 1F). Accordingly, females in DPN_pre_ and DPN_post_ groups exhibited decreased difference scores relative to vehicle and DPN_4h_ females (Fig. 1G). DPN did not affect difference scores in males. Vehicle and DPN_4h_ female difference scores were not different from males, despite having higher ANPs than males (Fig. 1F, G). These changes in heroin-cue EMR reveal a female-specific role for ERβ signaling during extinction memory consolidation in the BLA.

### Systemic ERβ agonism enhances heroin-cue EMR in both sexes

To investigate whether the EMR-enhancing effects of ERβ agonism are maintained with systemic administration, in Experiment 2 we administered DPN subcutaneously before cued extinction (Fig. 2A). ANPs, INPs, and intake were as reported in Experiment 1 (Fig. 2B (ANPs), 2C (intake: 2way RM ANOVA, main effect of sex, [*F*_1,29_ = 37.43, *p* < 0.0001]), and S3A (INPs: 2way RM ANOVA, main effect of sex, [*F*_1,29_ = 9.09, *p* = 0.0053])). During extinction, systemic DPN had no effect on ANPs in either sex, but females had greater ANPs than males (Fig. 2D, 2way ANOVA, main effect of sex, [*F*_1,27_ = 50.50, *p* < 0.0001]). Comparison of ANPs across the extinction session resulted in a significant time × sex interaction (Fig. 2E, 2way RM ANOVA, [*F*_3,87_ = 9.02, *p* < 0.0001]). On test, DPN improved EMR in both males and females, decreasing ANPs (Fig. 2F, 2way ANOVA, main effect of DPN, [*F*_1,27_ = 50.14, *p* < 0.0001]) and difference scores (Fig. 2G, 2way ANOVA, main effect of DPN, [*F*_1,27_ = 42.06, *p* < 0.0001]). Thus, ERβ agonism enhances EMR in both sexes, but does so presumably via distinct brain regions.

### Systemic ERβ agonism enhances heroin-cue EMR after abstinence

In Experiment 3, we administered DPN after protracted abstinence (Fig. 3A). ANPs and intake were as reported in Experiments 1 and 2 (Fig. 3B (ANPs), 3C (intake: 2way RM ANOVA, main effect of sex, [*F*_1,22_ = 19.03, *p* = 0.0002])). However, INPs did not differ between males and females in this Experiment (Fig. S3D). Following 21 days of abstinence, females had greater ANPs than males during cued extinction, while DPN was without effect (Fig. 3D, 2way ANOVA, main effect of sex, [*F*_1,20_ = 38.44, *p* < 0.0001]). Across the session, there was a main effect of sex (Fig. 3E, 2way RM ANOVA, [*F*_1,22_ = 40.58, *p* < 0.0001]). On test, DPN improved EMR in both males and females, decreasing ANPs (Fig. 3F, 2way ANOVA, main effect of DPN, [*F*_1,20_ = 17.59, *p* = 0.0004]) and difference scores (Fig. 3G, 2way ANOVA, main effect of DPN, [*F*_1,20_ = 26.08, *p* < 0.0001]). These findings show that ERβ-mediated improvements in EMR are dissociable from any effects of ERβ signaling during acute withdrawal and robust enough to counteract incubation of craving.

### Systemic ERβ agonism maintains enhancing effects on heroin-cue EMR following post-treatment abstinence

In Experiment 4 we tested DPN’s ability to maintain EMR improvements after abstinence (Fig. 4A). ANPs, INPs, and intake were as reported in Experiments 1 and 2 (Fig. 4B (ANPs), 4C (intake: 2way RM ANOVA, main effect of sex, [*F*_1,30_ = 14.99, *p* = 0.0005]) and S3G (INPs: 2way RM ANOVA, main effect of sex, [*F*_1,30_ = 10.22, *p* = 0.0033])). During cued extinction, DPN did not affect ANPs in either sex, but females had greater ANPs than males (Fig. 4D, 2way ANOVA, main effect of sex, [*F*_1,28_ = 41.96, *p* < 0.0001]). Comparison across the session resulted in a significant time × sex interaction (Fig. 4E, 2way RM ANOVA, [*F*_5,150_ = 25.30, *p* < 0.0001]). Following 21 days of abstinence, DPN improved EMR in both sexes, decreasing ANPs (Fig. 4F, 2way ANOVA, main effect of DPN, [*F*_1,28_ = 147.2, *p* < 0.0001]) and difference scores (Fig. 4G, 2way ANOVA, main effect of DPN, [*F*_1,28_ = 184.4, *p* < 0.0001]). While difference scores in DPN-treated subjects were not below zero, a single cued extinction session with the ERβ agonist significantly decreased drug-seeking 21 days after treatment and counteracted incubation of craving.

### Systemic ERβ agonism does not affect sucrose-cue EMR

In Experiment 5 we examined DPN’s impact on sucrose-cue EMR (Fig. 5A). Females and males had similar ANPs, but females had greater sucrose intake (g/kg) than males (Fig. 5B (ANPs), 5C (intake: 2way RM ANOVA, main effect of sex, [*F*_1,22_ = 10.56, *p* = 0.0037]) and S3J (INPs: 2way RM ANOVA, main effect of sex, [*F*_1,22_ = 8.48, *p* = 0.0081])). Neither sex nor DPN influenced ANPs during cued extinction (Fig. 5D, E), ANPs on EMR test (Fig. 5F), or difference scores (Fig. 5G).

### DPN has sex-specific impacts on anxiety-like behavior

During the open field test, females with prior heroin or sucrose self-administration had greater locomotor activity than males (Fig. S4A, D, 2way ANOVAs, main effects of sex, Heroin: [*F*_1,28_ = 12.5, *p* = 0.0015]; Sucrose: [*F*_1,20_ = 12.25, *p* = 0.0023]), but DPN did not affect general locomotor activity in either sex. DPN did suppress thigmotaxis in sucrose females (Fig. S4E, 2way ANOVA, DPN × sex interaction, Sucrose: [*F*_1,20_ = 5.81, *p* = 0.026]), and increased center time in all females (Fig. S4C, F, 2way ANOVAs, Heroin: DPN × sex interaction, [*F*_1,28_ = 5.774, *p* = 0.0015]; Sucrose: main effect of DPN, [*F*_1,20_ = 14.27, *p* = 0.0012], post-hoc *p* < 0.05 in females only), indicative of acute anxiolytic effects. Comparisons between vehicle-sucrose and vehicle-heroin subjects show that heroin withdrawal increased general locomotor activity, increased thigmotaxis, and decreased center time relative to sucrose, with sex differences in locomotion and thigmotaxis (Fig. S4I–G, 2way ANOVAs, Locomotor: main effects of heroin, [*F*_1,24_ = 5.28, *p* = 0.031] and sex [*F*_1,24_ = 11.25, *p* = 0.0026]; Thigmotaxis: main effects of heroin, [*F*_1,24_ = 18.73, *p* = 0.0002] and sex [*F*_1,24_ = 9.04, *p* = 0.0061], Center Time: main effect of heroin, [*F*_1,24_ = 4.99, *p* = 0.035]).

### ERβ is sex-specifically expressed in the basal portion of the BLA

**Fig. 6:**
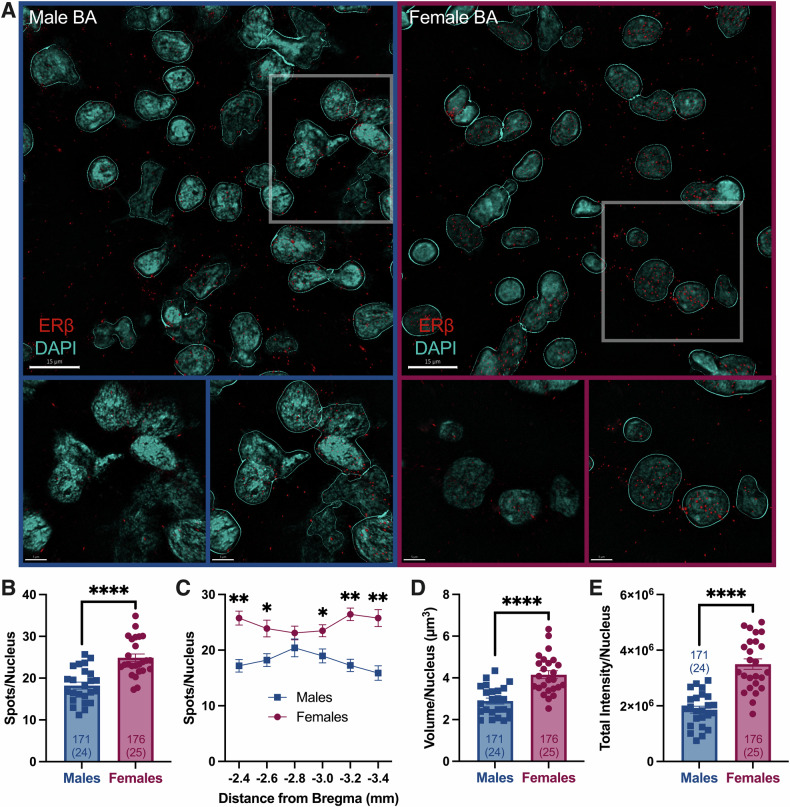
Females have greater ERβ expression in the BA than males. **A** Representative confocal photomicrographs from male (left) and female (right) basal (BA) portions of the BLA. Lower magnification Imaris-processed images are shown on top (scale bars = 15 µm). The gray box depicts the higher magnification images shown below (scale bars = 5 µm): unprocessed (left) and Imaris-processed (right). **B** Number of ERβ spots in the BA adjusted for number of nuclei. ERβ spots were greater in females than males. **C** Distribution of ERβ spots along the anterior-posterior axis of the BA. ERβ spots were greater in females, particularly in the anterior and posterior aspects of the BA. ERβ spots did not significantly differ across the BA in either sex. **D** ERβ spot volume in the BA adjusted for number of nuclei. ERβ spot volume was greater in females than males. **E** Total intensity of voxels within ERβ spots in the BA adjusted for number of nuclei. ERβ spot intensity was greater in females than males. Data shown as mean ± SEM with individual averages for each subject shown. Image and subject *n’*s are shown within columns as Images (Subjects). *p < .05, **p < .01, ****p < .0001. BLA, basolateral amygdala; BA, basal portion of the BLA.

Analysis of IHC did not detect any effects of Veh versus DPN or Heroin versus Sucrose on ERβ expression (Figure S5), so data are shown collapsed across these groups (Fig. 6). Representative photomicrographs, with and without Imaris-processing, are shown in Fig. 6A. In the basal BLA (BA), there were more ERβ spots per nucleus in females than males (Fig. 6B, nested *t* test, t[47] = 5.45, *p* < 0.0001), occurring throughout most of the BA (Fig. 6C). There were no expression differences between hemispheres (Fig. S6). ERβ spot volume and total intensity of voxels within spots were also greater in females (Fig. 6D, E, nested *t* tests, Volume: *t*[47] = 5.35, *p* < 0.0001, Intensity: *t*[47] = 6.82, *p* < 0.0001). No differences in ERβ expression were detected in the lateral BLA (LA), the central amygdala (CeA), the medial amygdala (MeA), or paraventricular nucleus of the hypothalamus (PVN, Fig. S7). ERβ expression was below the detection threshold in the accessory basal BLA (AB).

## Discussion

Together, our findings establish a novel role for ERβ in EMR of heroin-conditioned cues. Specifically, ERβ agonism during cued extinction reliably enhanced heroin-cue EMR across several experiments, demonstrating potential clinical utility as an adjunct to CET. This agrees with our previous study showing inhibition of E2 signaling impairs EMR [48] and reinforces the established contributions of E2 signaling to fear EMR [43–47]. This study expands our understanding of the interactions between sex, central E2 signaling, cue-induced drug seeking, and EMR in several ways.

### ERβ agonism in the BLA enhances consolidation of extinction memories in females

ER antagonism in the BLA results in sex-specific impairments in heroin-cue EMR; occurring via ERα in males but ERβ in females [48]. As predicted, the ERβ agonist (DPN) administered directly into the BLA improved EMR in females only (Fig. 1). Therefore, ERβ’s impacts on heroin-cue EMR in females are bidirectional (can enhance or impair), as in fear EMR [43–47]. While speculative, sex differences in ERβ expression in the BA (females > males), may underly this behavioral distinction (Fig. 6), though downstream effectors could also contribute [38, 55]. For example, females have greater expression of the type 1 metabotropic glutamate receptor (mGluR1) in the BLA than males [56], and long-term depression in the BLA is dependent on mGluR1 in males (untested in females) [57]. Since membrane-associated ERs functionally couple with mGluRs to signal [38], this difference could drive sex divergent effects, like the impact on EMR here.

We also determined the specific memory process impacted by ERβ in heroin-cue EMR. DPN administered into the BLA of females immediately after the cued extinction session (during extinction memory consolidation) also enhanced heroin-cue EMR, but not when given outside of the consolidation window (Fig. 1). Therefore, ERβ signaling in the BLA improves heroin-cue EMR by enhancing extinction memory consolidation in females. This finding is consistent with other studies reporting that E2 enhances memory consolidation (reviewed in [31, 58]), including fear EMR [46]. Though the functional effects of E2 on memory are largely similar between the sexes, convergent sex differences [59] have started to emerge at the cellular and molecular level [60–62]. In addition, most studies have focused on systemic or hippocampal E2 manipulations, with regions like the BLA remaining largely unexplored. The specific molecular pathways and cell types involved are beyond the scope of this study, but other publications have reported numerous factors that impact extinction memory consolidation in the BLA (e.g., L-type calcium channels [63], NMDA receptors [64, 65], and GABAergic, cholinergic, or serotonergic signaling [66, 67], several of which could be impacted by ERβ.

### Systemic ERβ agonism improves heroin-cue EMR in both sexes

Next, to enhance translational applicability, we tested whether systemic DPN administration would have similar effects on EMR. Even though EMR in males was unaffected by BLA ERβ agonism, we included males since we hypothesized that DPN could affect other brain regions to alter EMR. Interestingly, systemic DPN administration improved heroin-cue EMR in both sexes during the early abstinence period (Fig. 2). This convergent sex effect [59] indicates that an additional region(s) is involved in ERβ signaling and heroin-cue EMR in males.

Our findings that DPN does not impact cued extinction behavior and improves cued EMR (anti-drug-seeking) agrees with prior studies of E2 signaling when drug is not available [68, 69] (though one only had effects in food restricted, not sated, subjects [67]). In one study using a traditional extinction paradigm (no drug cues), E2 promoted heroin EMR between the first two days of extinction [69], similar to the enhancement in contextual fear EMR noted by another group [70]. However, prior studies have found that E2 signaling, sometimes via ERβ, can instead promote various addiction-like behaviors [71–74], though this may differ significantly between stimulants and opioids [69, 75]. Here, our seemingly conflicting results can be attributed to differences in administration timepoints (during active drug use versus during extinction) among other protocol differences (ovariectomy, drug class, drug-primed reinstatement); however, evaluating potential pro-drug seeking effects are an important consideration prior to clinical use.

Notably, this finding of DPN-enhanced EMR is specific for heroin-conditioned cues, as sucrose-cue EMR is robust regardless of DPN (Fig. 5). We posit that the lack of effect on sucrose-cue EMR does not reflect a difference in ERβ signaling. Rather, we hypothesize that neuroadaptations following opioid use cause perseverative responding that is resistant to extinction (poor EMR), while “natural” rewards are more amenable to extinction without intervention. In fact, there are numerous differences in the processing of “natural” versus drug rewards in the brain [76]. This is supported by our finding of a floor effect on sucrose-cue EMR, which could limit the detection of possible ERβ-mediated changes. This may be addressed by shortening the cued extinction session, allowing better detection of an enhancement in sucrose-cue EMR by DPN.

### Systemic ERβ agonism modulates anxiety-like behavior in females

In rodents, locomotor hyperactivity and anxiety-like behavior are common symptoms of acute heroin withdrawal [8]. We tested these behaviors in an open field test, conducted 24 hours after the last self-administration session during early heroin withdrawal. On this task, time spent in the center and thigmotaxis are common indices of anxiety-like behavior [77]. In vehicle-treated subjects, heroin self-administration increased locomotor activity, increased thigmotaxis, and decreased time spent in the center relative to sucrose (Figure S4). These findings are similar to results from our lab [78] and others [79, 80]. Additionally, in agreement with several prior studies, females had greater locomotor activity than males and exhibited an anxiolytic response following DPN [52, 81–83] (however, also see [84, 85]). Here, ERβ agonism did not impact anxiety in males [53], although E2 has decreased anxiety-like behavior in males previously [86]. Our findings reinforce that the mechanism of anxiolysis does not appear to involve ERβ in males, in contrast to females.

### ERβ-mediated improvements in heroin-cue EMR are long-lasting

Forced abstinence “incubates” cued drug craving [27, 28] and mimics delays between cessation of drug use and treatment initiation seen clinically [87]. Following a period of protracted abstinence before cued extinction/treatment, DPN still improved EMR in both sexes, but interesting patterns of responding emerged (Fig. 3). Vehicle females had greater responding during test than all other groups, but vehicle males had the poorest difference scores. This divergence supports our decision to assess both ANPs and difference scores as measures of EMR. A large decrease in ANPs between extinction and test results in a favorable difference score, even when unadjusted ANPs are relatively high (vehicle females). This difference may also reflect an effect of frequency on learning: since vehicle females had greater ANPs, they earned more cue exposures during EMR test than males. Therefore, females had increased opportunities to extinguish the heroin-cue association, which is reflected by a relatively large decrease in ANPs between extinction and test.

While ERβ agonism enhances EMR in the short-term, remarkably, a single DPN injection prior to cued extinction still enhanced EMR when a 3-week abstinence period was introduced between cued extinction and EMR test (Fig. 4). While difference scores were not below zero, DPN substantially blunted the effects of incubation on cued heroin seeking. Therefore, ERβ agonism has durable effects on EMR after a single treatment. While incubation of opioid craving, particularly as a clinical phenomenon, is controversial [28, 88], it does not diminish the potential translational applicability of ERβ agonists as pharmacologic adjuncts for CET. As we show in other experiments, the primary effect of DPN is to enhance the broader, clinically-relevant process of EMR [16, 17], not to counteract incubation directly.

To further reduce drug-seeking after extended abstinence, the efficacy of ERβ agonists to enhance heroin-cue EMR in this model could be evaluated in combination with clinically-relevant modifications, including multiple cued extinction/CET sessions and the addition of medications that reduce opioid craving, like methadone.

### Females have greater ERβ expression than males in the BA

Measurement of ERβ expression has been fraught with antibody problems [39], likely contributing to poor interstudy replicability and contrasting results [40–42]. For example, while both studies detected ERβ in the BLA, they conflicted significantly on expression in the LA [41, 42]. Problematically, inconsistencies have also been reported in mRNA analyses [89–92] and between mRNA and protein studies. The low inclusion of males and frequent use of ovariectomized females further limit assessment of ERβ expression.

More recently, ERβ expression has been measured using a transgenic reporter line expressing ERβ-EGFP [93]. This tool is currently restricted to mice, and patterns/levels of ERβ expression may not be conserved in rats. Here, we used a rigorously validated anti-ERβ monoclonal antibody [39, 54] to determine ERβ expression in several brain regions of gonadally intact male and female rats. We found that females have greater ERβ expression than males in the BA (Fig. 6), which could underly the convergent sex effect of ERβ agonism on EMR. No other regions examined herein (LA, CeA, MeA, PVN) had sex-differential ERβ expression (Fig. S7).

## Conclusions

In this study, we demonstrated that ERβ signaling during cued extinction enhances heroin-cue EMR across several translational experimental designs. This effect is present in both sexes when administered systemically (convergent sex effect), but ERβ agonism in the BLA only enhances EMR in females, specifically by promoting extinction memory consolidation. This may be driven by increased ERβ expression in the female BA, where ERβ is uniquely elevated compared to males. While estrous cycle phase did not impact heroin-cue EMR [48], it could affect other measures included herein, like ERβ expression. While beyond the scope of the present study, future investigations should examine the impacts of estrous cycle phase, molecular mechanisms underlying sex-specific expression of ERβ in the BA, downstream molecular consequences, region(s) that mediate ERβ’s impact on EMR in males. Furthermore, the contributions of other ERs (ERα, GPER) should also be explored, as E2 may impact EMR via several mechanisms. Given our primary goal, to test ERβ’s contributions to heroin-cue EMR in a preclinical model of OUD/CET, the existence of clinically viable ERβ agonists already under investigation (e.g., Prinaberel and Erteberel) may facilitate translation of this approach into clinical trials.

### Supplementary information

Supplemental Methods

Supplementary Tables

## Data Availability

Detailed materials and methods and complete statistical analyses are included in the supplemental materials. Additional data are available upon request to the corresponding authors.
