# Supplementary material for: Estrogen receptor beta signaling enhances extinction memory recall for heroin-conditioned cues in a sex- and region-specific manner
Source: Transl Psychiatry. 2024 Jul 12;14:283. doi: 10.1038/s41398-024-03001-y (PMC11245532; doi:10.1038/s41398-024-03001-y)
Supplement: Supplementary file 1 — Supplemental Methods [file 41398_2024_3001_MOESM1_ESM.pdf]

*Supplement for *Translational Psychiatry*

Supplemental Methods:

<u>*Subjects & Surgeries*</u>:

A total of 158 male and female Wistar rats (8-9 weeks old on arrival, Envigo, Indianapolis, IN) were used, with 4 subjects excluded from final analyses due to catheter failure or missed cannula placement. Each experiment was conducted across at least two independent cohorts to ensure replicability. Rats were pair-housed on a reverse 12:12 light:dark cycle with food and water provided *ad libitum* for the duration of the study.

Following a 7-day acclimation period, subjects in Experiments 1-4 had an intravenous catheter (SAI Infusion Technologies, Lake Villa, IL) implanted for heroin self-administration. Catheters were inserted 4cm into the right jugular vein and secured with silk sutures. The opposite side of the tubing ran subcutaneously, connecting to a port on the back below the shoulder blades. At the time of surgery, the port was locked with 0.05mL (equivalent to the catheter dead volume) heparinized glycerol (500 USP/mL heparin in 50/50 v/v glycerol/normal saline).

Rats in Experiment 1 also had bilateral cannulas (6.5 mm extension from base, P1 Technologies, Roanoke, VA) stereotactically implanted for pharmacologic manipulation of ERs in the BLA (coordinates for cannula placement relative to bregma: AP: –2.5mm, ML: ±4.8mm, DV: –6.5mm). Injectors (P1 Technologies) projected 2 mm beyond the end of the cannula, such that the target infusion location was at DV: –8.5mm. Cannulas were inserted with cannula holders on a digital stereotax (David Kopf

Instruments, Tujunga, CA) and secured; initially with Geristore resin-ionomer (DenMat, Lompoc, CA) and then with dental acrylic (Ortho-Jet BCA; Lang Dental, Wheeling, IL) to form a headcap.

All surgeries were performed under isoflurane anesthesia (4% induction, 1.5-2.5% maintenance). For analgesia, ketorolac (5.0mg/kg, Sigma-Aldrich, St. Louis, MO) or meloxicam (1.0mg/kg, VetOne, Paris, France) was administered subcutaneously on the day of surgery and meloxicam tablets (0.5mg/tablet; Bio-Serv, Flemington, NJ) were provided for two post-surgical days.

Drugs & Infusions:

Heroin (NIDA Drug Supply Program, Rockville, MD) was dissolved in 0.9% sterile saline to 200mg/250mL. Sucrose pellets (45mg; Bio-Serv, Flemington, NJ) were placed in hoppers.

The ERβ agonist (diarylpropionitrile, DPN, MedChemExpress, Monmouth Junction, NJ) was dissolved in DMSO and diluted with saline. DPN was administered intracranially (IC, Exp. 1) or subcutaneously (SC, Exps. 2-5). For IC administration, DPN was weighed and dissolved in DMSO to form a stock solution (400ng/mL). Just prior to administration, 975μL of 0.9% saline (37 °C) was slowly added to 25μL of the stock solution for a final concentration of 10pg/μL in 2.5% v/v DMSO. This solution was infused (1μL at a rate of 0.2μL/min), yielding a dose of 10pg/hemisphere[48]. DPN has 70-fold greater affinity for ERβ over ERα[49] and is specific for ERβ at this low dose[50]. For SC administration, DPN was weighed and dissolved in DMSO then diluted with 0.9% saline (37 °C) for a final concentration of 1mg/mL in 50% v/v DMSO. This solution was

injected SC (1mL per 1kg body weight), yielding a dose of 1mg/kg[51,52]. Given the behavioral effects present at the doses used, the previous publications on these doses (cited above), and for the sake of experimental efficiency, we did not test other doses in the present study.

Heroin/Sucrose Self-Administration:

Just prior to the first self-administration session, the heparinized glycerol locking solution was pulled back and catheter patency was checked via blood return. Subjects without blood return (~ 7%) had patency checked with Brevital (Methohexital; Par Pharmaceutical, Chestnut Ridge, NY) before proceeding to self-administration. Two recatheterizations were performed based on failure of the Brevital check prior to beginning self-administration.

Rats self-administered heroin (Exps. 1-4) or sucrose (Exp. 5) via nose pokes for 6hrs per day for 8 consecutive days (days 14-21). Self-administration operant chambers (30 x 20 x 20cm) were enclosed in sound-attenuating cabinets with ventilation fans (Med Associates, Fairfax, VT). Responses in the chambers were recorded on MED PC-V software (Med Associates). Each chamber had two nose poke receivers, white stimulus lights above each receiver, a house light, and tone generator (78dB, 4.5kHz). Infusions were delivered by metal leashes magnetically connected to the catheter port on the rat (SAI Infusion Technologies, Lake Villa, IL). The leash was connected to a weighted swivel apparatus (SAI Infusion Technologies) suspended above the chamber to allow for unrestricted movement.

Active nose pokes (ANPs) led to delivery of heroin (40μg) or sucrose (45mg) with a 5-sec light and tone stimulus (reward-associated cue). This was followed by a 20-sec timeout period, during which active and inactive responses were recorded, but no reward or cues were delivered. Inactive nose pokes (INPs) had no programmed outcome. The first four days were on a fixed ratio (FR) 1 reinforcement schedule, requiring one ANP to obtain the reward. The last four days were on an FR3, requiring three operant responses for reward delivery. Small wood dice (16 x 16mm) were present in each operant chamber during self-administration of all experiments to discourage heroin-driven injurious chewing behaviors (chewing on paws).

Cued Extinction & Extinction Memory Recall:

After 8 days of self-administration (days 14-21), subjects underwent a 1hr (Exps. 1-3, 5) or 6hr (Exp. 4) cued extinction session. This session occurred the following day (day 22 in Exps. 1, 2, 4, and 5) or after 21 days of home cage abstinence (day 43 in Exp. 3). During this session, ANPs (FR1) led to presentation of the reward-associated light+tone cue without heroin/sucrose delivery. DPN was administered prior to (Exps. 1-5) the cued extinction session (preparation and doses detailed above). In Experiment 1, a subset of females received DPN at other timepoints: immediately after the extinction session (within the consolidation window) or 4hrs later (outside of the consolidation window).

Following cued extinction, rats were tested for extinction memory recall (EMR). The EMR test occurred the day after cued extinction (day 23 in Exps. 1, 2, and 5; day 44 in Exp. 3) or after 21 days of home cage abstinence (day 44 in Exp. 4). Conditions

during the 1hr session were the same as cued extinction (ANPs resulted in presentation of the conditioned cues only), except no DPN was administered.

Open Field Test:

Subjects in Experiments 2 and 5 were assessed for locomotor activity and anxiety-like behavior immediately after the cued extinction session (while DPN was active). The test was conducted for 5min on a round open field (100cm diameter) with 25cm high walls. The field was illuminated by a light directly above the center of the apparatus such that the center area was bright, but the areas near the walls were dim. The apparatus was cleaned with 10% ethanol after each subject to eliminate odor cues. Behavior was recorded and tracked using EthovisionXT (v.11.5, Noldus, Wageningen, Netherlands). Total distance traveled, time spent in the center of the field (away from the walls), and distance traveled around the perimeter walls (thigmotaxis) were automatically scored.

Tissue Preparation:

Thirty minutes after the EMR test, subjects were rapidly decapitated, and brains were extracted and flash-frozen in isobutane and dry ice. Brains (Exps. 1, 2, & 5) were sliced on a cryostat at 20μm, with concurrent verification of cannula placements (Exp. 1). Sections from Experiments 2 and 5 were slide-mounted (Superfrost Plus, Fisher Scientific, Waltham, MA) and processed via immunohistochemistry (IHC) as detailed below.

Immunohistochemistry:

Methods and antibody concentrations were optimized in small batches. Once optimized, IHC was performed in several batches with mixed subject samples from within and between cohorts to control for inter-batch variability. Each batch included positive and negative controls.

Slide-mounted slices were fixed and permeabilized using acetone (chilled at -20°C prior) for 10min at -20°C. Samples were removed from acetone and air-dried at room temperature for 10min prior to being immersed in a new well of chilled acetone for an additional 10 min at -20°C. Slides were removed and air dried for 10-20min at room temperature, until completely dry.

Slides were rinsed with room temperature TBS for 10min, 3 times. Slides were dried for 10-20min at room temperature until completely dry. A barrier was drawn around each sample with a hydrophobic barrier pen (ImmEdge, Vector Laboratories, Newark, CA) and allowed to dry. Samples were rehydrated in TBS for 10min at room temperature. Blocking buffer was prepared by adding normal goat serum (Jackson ImmunoResearch, West Grove, PA) to TBS to yield a 10% normal goat serum solution. Enough blocking buffer was prepared to be used for the blocking step and to dilute the primary antibody. The blocking buffer was applied onto the slides with a dropper, enough to cover each slice, and left undisturbed for 1hr in a humidified chamber (damp filter paper in a covered metal container) at room temperature.

The primary antibody (mouse anti-ERβ monoclonal PPZ0506; Invitrogen, Waltham, MA) was diluted in blocking buffer at a ratio of 1:200. After tapping off the blocking buffer, the primary solution was applied onto the slides with a dropper, enough

to cover each slice. Slides were left undisturbed overnight (16hrs) in a humidified chamber at 4°C. The next day, slides were rinsed with TBS for 10min, 3 times. The secondary antibody (goat anti-mouse CF594, Biotium, Fremont, CA) was diluted in TBS at a ratio of 1:250. The secondary solution was applied to the rinsed slides as before. Samples incubated at room temperature for 1hr in the covered humidified chamber. Slides were rinsed with TBS for 10min, 3 times. Next, DAPI (Biotium) was applied to each slide as before and incubated for 30sec before being tapped off. Slides were dried for 15-30min away from light and coverslipped using Prolong Glass (Invitrogen) and coverslips (Corning, no. 1½, Corning, NY). Samples were imaged between 24 and 72hrs later.

Confocal Microscopy & Image Analysis:

All images were acquired using a Leica (Wetzlar, Germany) Stellaris 5 confocal microscope at a resolution of 1024 x 1024 using a 63x oil objective and 1.5x optical zoom at a speed of 400 Hz (unidirectional X). The Z-step was set to 0.25μm, the pinhole was 1 AU, and a frame average of 2 was applied. Together, this resulted in a pixel dwell time of 1.58 μs and a frame rate of 0.194/s with a final image size of 123.13 x 123.13μm and a pixel size of 120.36 x 120.36nm. Intensity for the 405 and 594nm lasers were 2.5% and 5%, respectively. Gain for both channels was set to 15%. Following acquisition, Leica "Lightning" deconvolution was automatically applied to each image (parameters automatically determined by speed and resolution).

All images were imported into Imaris (v. 9.0, Oxford Instruments, Abingdon, United Kingdom) and processed by an experimenter blinded to the experimental

conditions. In the Display Adjustment, all channels were reset to ensure uniform color display. Next, the following processing steps occurred: background subtraction (5μm) for the 405/DAPI channel and baseline subtraction (1500) for the 594/ERβ channel. To classify nuclei (DAPI), the "Surfaces" tool was used. The surface detail grain size was set to .750μm. Threshold for detection, based on absolute intensity, was manually assigned for each image by a blinded investigator. Split touching objects was enabled with a seed point diameter of 8.00μm. Seed points were then filtered by quality, requiring a score of ≥15. Finally, surfaces were filtered by number of voxels, requiring ≥100. Similarly, the "Spots" tool was used to count ERβ. Region growing was enabled to allow for detection of different spot sizes. Estimated XY diameter and Z diameter were both .250μm. Background subtraction and modeling of the PSF along the Z-axis were both enabled. Spots were filtered by quality, requiring a score of ≥1000. Region growing was dictated by absolute intensity using automatic threshold parameters. The diameter was determined based on the region volume. Measures included the total number of spots in the image, summated volumes for all spots, and summated total intensities for all voxels contained within spots. Each measure was normalized by dividing its value by the number of nuclei in the image.

Data Analysis:

To control for individual differences in levels of operant responding when assessing EMR, we calculated a difference score ($ANPs_{EMR\ Test}$ *[total]* – $ANPs_{Extinction}$ *[average/hour]*) (48). This allows for direct, standardized, within-subjects comparisons of responses between cued extinction and the EMR test across experiments. Difference

scores are one metric to assess EMR by adjusting for individual differences in responding, but they should be interpreted in the context of other data (like raw ANP responding), which is also provided. While several calculations of the difference score could be performed using total ANPs from the extinction session or ANPs only from a selected time period of the session (opposed to the average hourly responses, as used here), the interpretation largely does not change.

All analyses and graphs were produced using Prism software (v.10, GraphPad). Dependent measures (nose pokes, difference scores, intake, locomotor activity, ERβ expression) were analyzed using t-tests (paired/unpaired ± nesting) or analyses of variance (ANOVAs; 1 or 2way ± repeated measures [RM] and/or nesting; with Geisser-Greenhouse corrections). For all ANOVAs, the between subjects' independent variables were sex (male/female) and treatment during extinction (Veh/DPN). Holm-Šídák's post-hoc comparisons were used as indicated. The significance was set at $\alpha=0.05$ throughout. Complete statistics are included in Tables S1-S7. INPs during cued extinction and EMR test did not differ by sex or treatment in any of the Experiments (Table S8). Additional behavioral data (INPs during self-administration, heroin infusions/sucrose deliveries, and direct comparisons of ANP responding between extinction and EMR test) are shown in Figures S2 (Exp. 1) and S3 (Exps. 2-5).

Supplemental Figures:

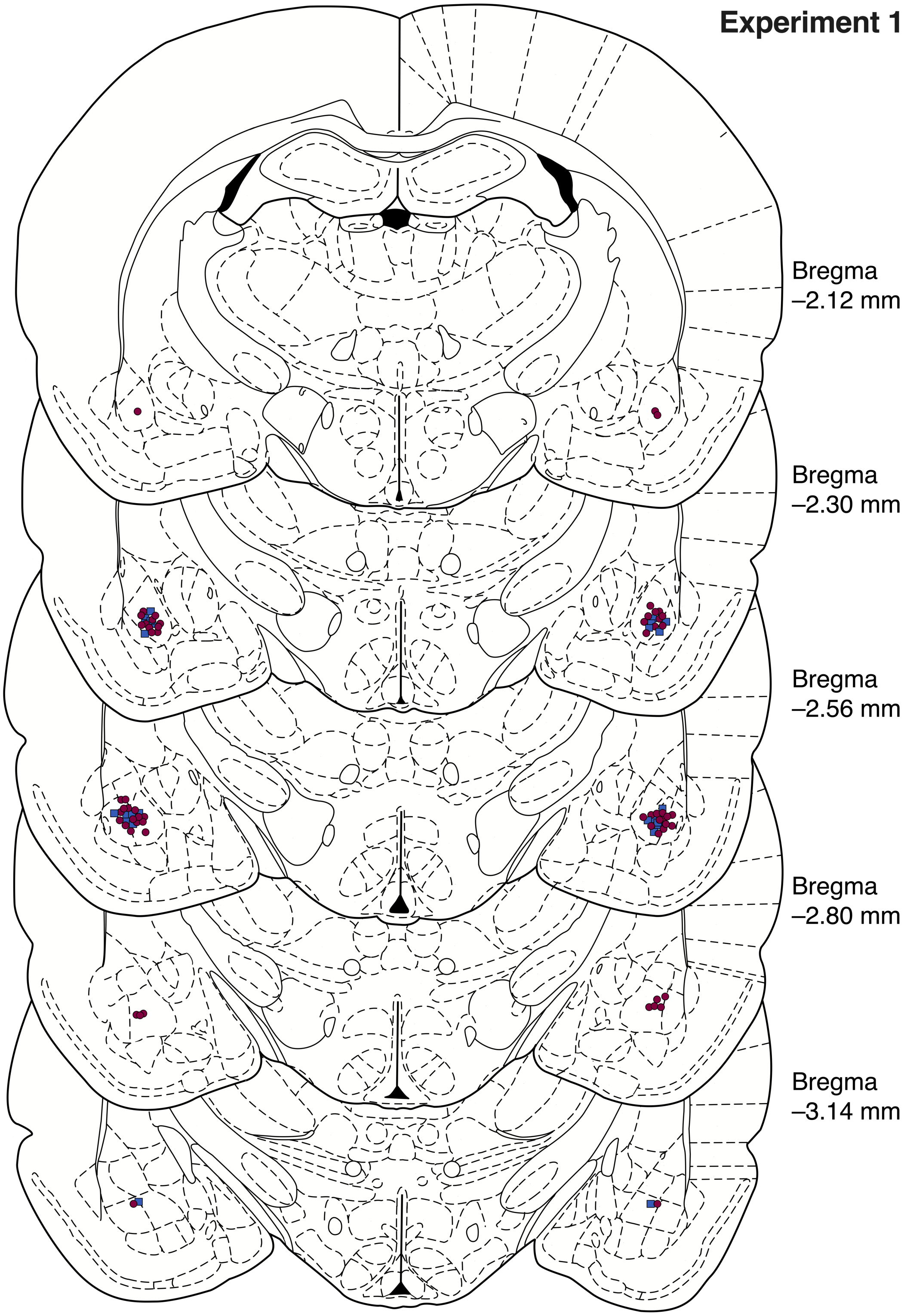

Figure S1. Enlarged cannula placements from Experiment 1.

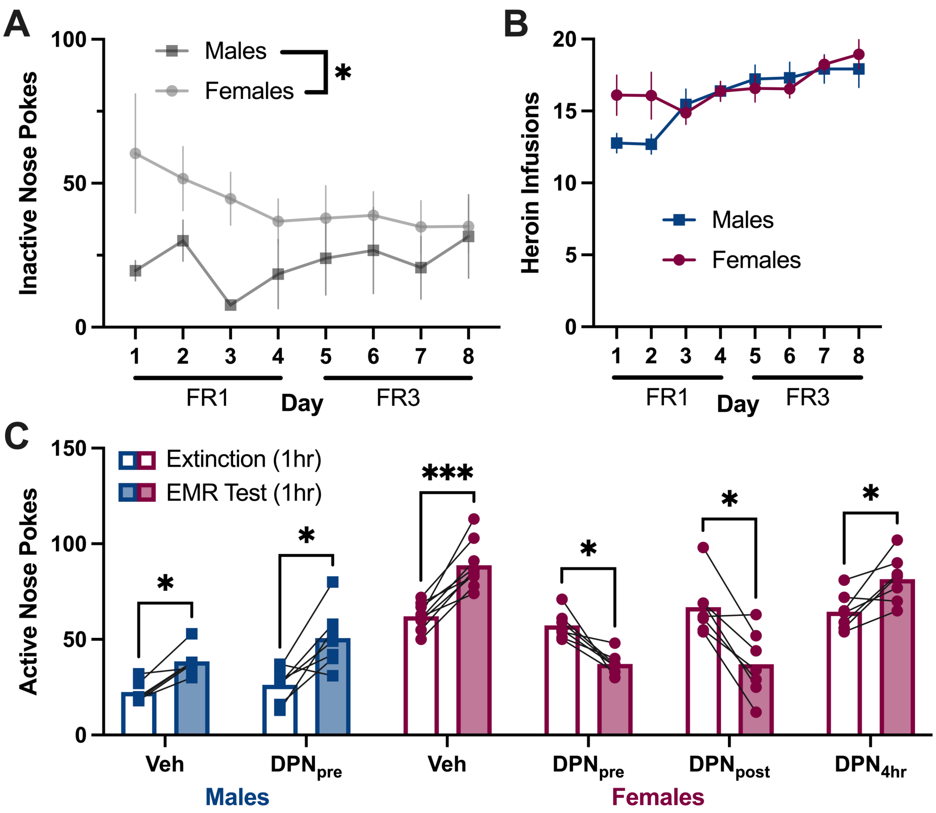

Figure S2. Experiment 1: Additional behavioral parameters. A. INPs during heroin self-administration. Females had greater INPs than males (main effect of sex). B. Heroin infusions during heroin self-administration. Infusions did not differ between males and females. C. Direct within-subject comparisons of total ANPs during the 1hr cued extinction session and 1hr EMR test (paired t-tests). All data are shown as mean ±SEM. *$p < .05$, ***$p < 0.001$. ANPs, active nose pokes; DPN, diarylpropionitrile (ERβ agonist); EMR, extinction memory recall; FR, fixed ratio; INPs, inactive nose pokes.

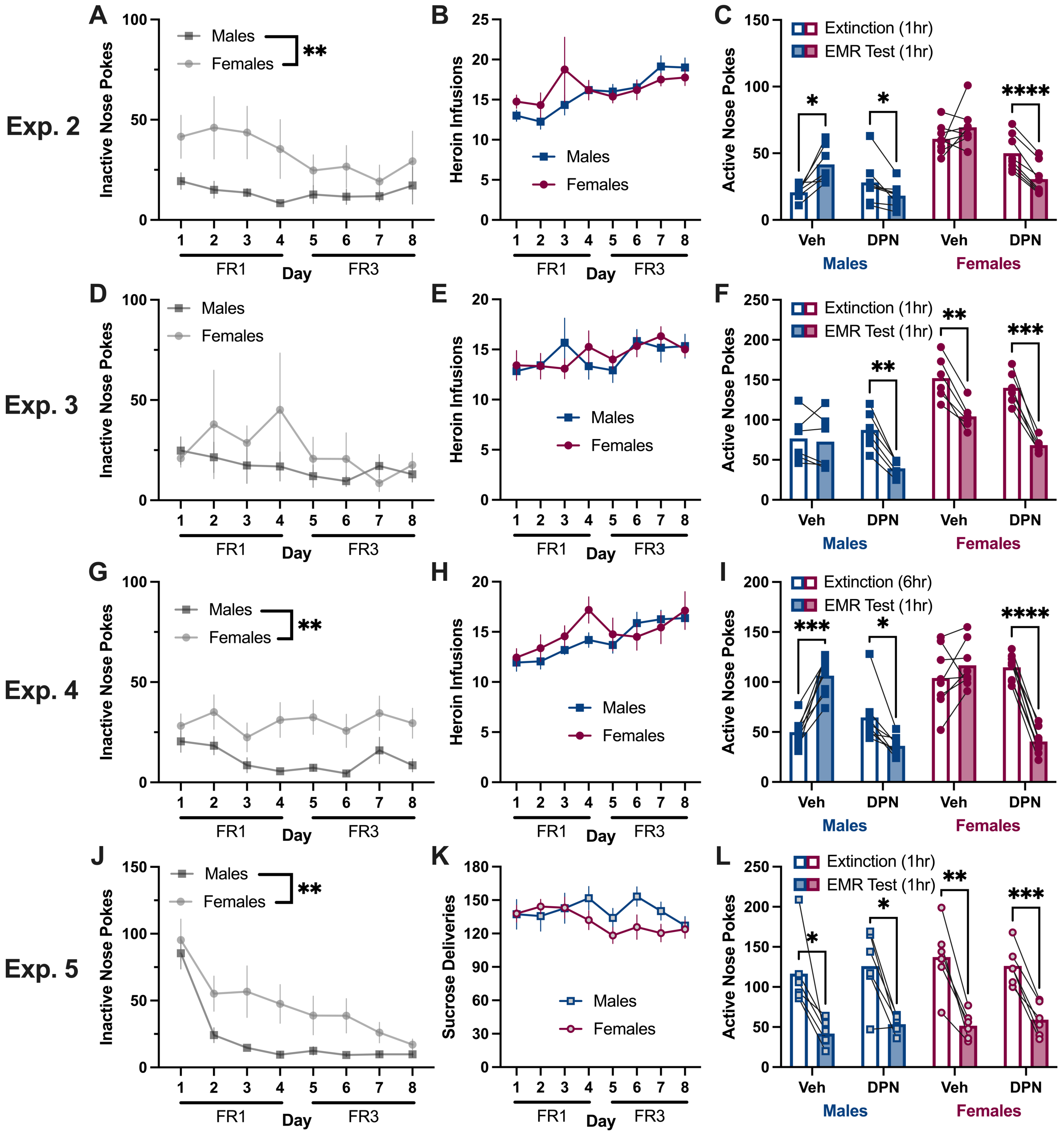

Figure S3. Experiments 2-5: Additional behavioral parameters. **A-C: Experiment 2**. A. INPs during heroin self-administration. Females had greater INPs than males (main effect of sex). B. Heroin infusions during heroin self-administration. Infusions did not differ between males and females. C. Direct within-subject comparisons of total ANPs during the 1hr cued extinction session and 1hr EMR test (paired t-tests). **D-F: Experiment 3**. D. INPs during heroin self-administration. Females and males did not differ in INP responding. E. Heroin infusions during heroin self-administration. Infusions did not differ between males and females. F. Direct within-subject comparisons of total ANPs during the 1hr cued extinction session and 1hr EMR test (paired t-tests). **G-I: Experiment 4**. G. INPs during heroin self-administration. Females had greater INPs

than males (main effect of sex). H. Heroin infusions during heroin self-administration. Infusions did not differ between males and females. I. Direct within-subject comparisons of total ANPs during the 6hr cued extinction session and 1hr EMR test (paired t-tests). **J-L: Experiment 5**. J. INPs during sucrose self-administration. Females had greater INPs than males (main effect of sex). K. Sucrose deliveries during sucrose self-administration. Deliveries did not differ between males and females. L. Direct within-subject comparisons of total ANPs during the 1hr cued extinction session and 1hr EMR test (paired t-tests). All data are shown as mean ±SEM. $^*p < .05$, $^{**}p < .01$, $^{***}p < 0.001$, $^{****}p < 0.0001$. ANPs, active nose pokes; DPN, diarylpropionitrile (ERβ agonist); EMR, extinction memory recall; FR, fixed ratio; INPs, inactive nose pokes.

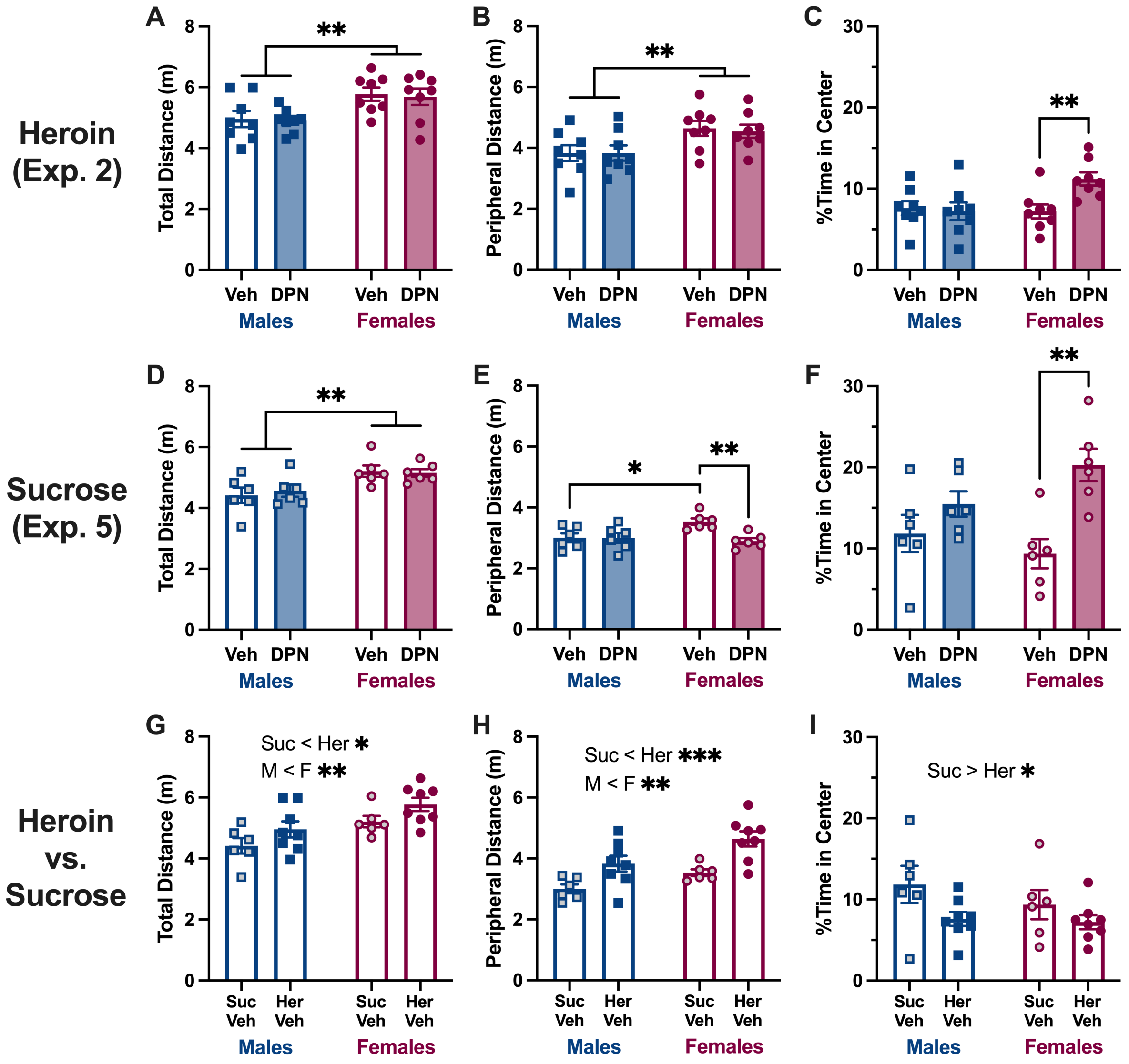

Figure S4. DPN decreases anxiety-like behaviors in females but does not impact locomotor activity in an open field. **A-C. Experiment 2**. A. Total distance (m) traveled during the open field task in heroin self-administering subjects. DPN did not alter locomotor activity, but females were more active than males (ME of sex). B. Distance traveled (m) in the peripheral arena (thigmotaxis). DPN did not alter thigmotaxis, but females had greater peripheral locomotor activity than males (ME of sex). C. Percent of total time spent in the center of the open field. Veh males and females spent similar amounts of time in the center. DPN increased center time in females only. **D-F. Experiment 5**. D. Total distance (m) traveled during the open field task in sucrose self-administering subjects. DPN did not alter locomotor activity, but females were more active than males (ME of sex). E. Distance traveled (m) in the peripheral arena (thigmotaxis). Veh females had greater peripheral activity than males, but DPN decreased thigmotaxis in females. F. Percent of total time spent in the center of the open field. Veh males and females spent similar amounts of time in the center. DPN

increased center time (ME of DPN), but the post-hoc was only significant in females. **G-I. Experiments 2 and 5** (data reproduced from Veh groups of A-F above) G. Total distance (m) traveled during the open field task in heroin and sucrose self-administering subjects. Prior heroin self-administration increased locomotor activity (ME of heroin) and females were still more active than males (ME of sex). H. Distance traveled (m) in the peripheral arena (thigmotaxis). Heroin increased thigmotaxis in both sexes (ME of heroin) and females still exhibited greater peripheral activity than males (ME of sex). I. Percent of total time spent in the center of the open field. Heroin decreased center time (ME of heroin). All data are shown as mean ±SEM. *$p < .05$, **$p < .01$, ***$p < .001$. DPN, diarylpropionitrile (ERβ agonist); Her, heroin; ME, main effect; Suc, sucrose; Veh, vehicle.

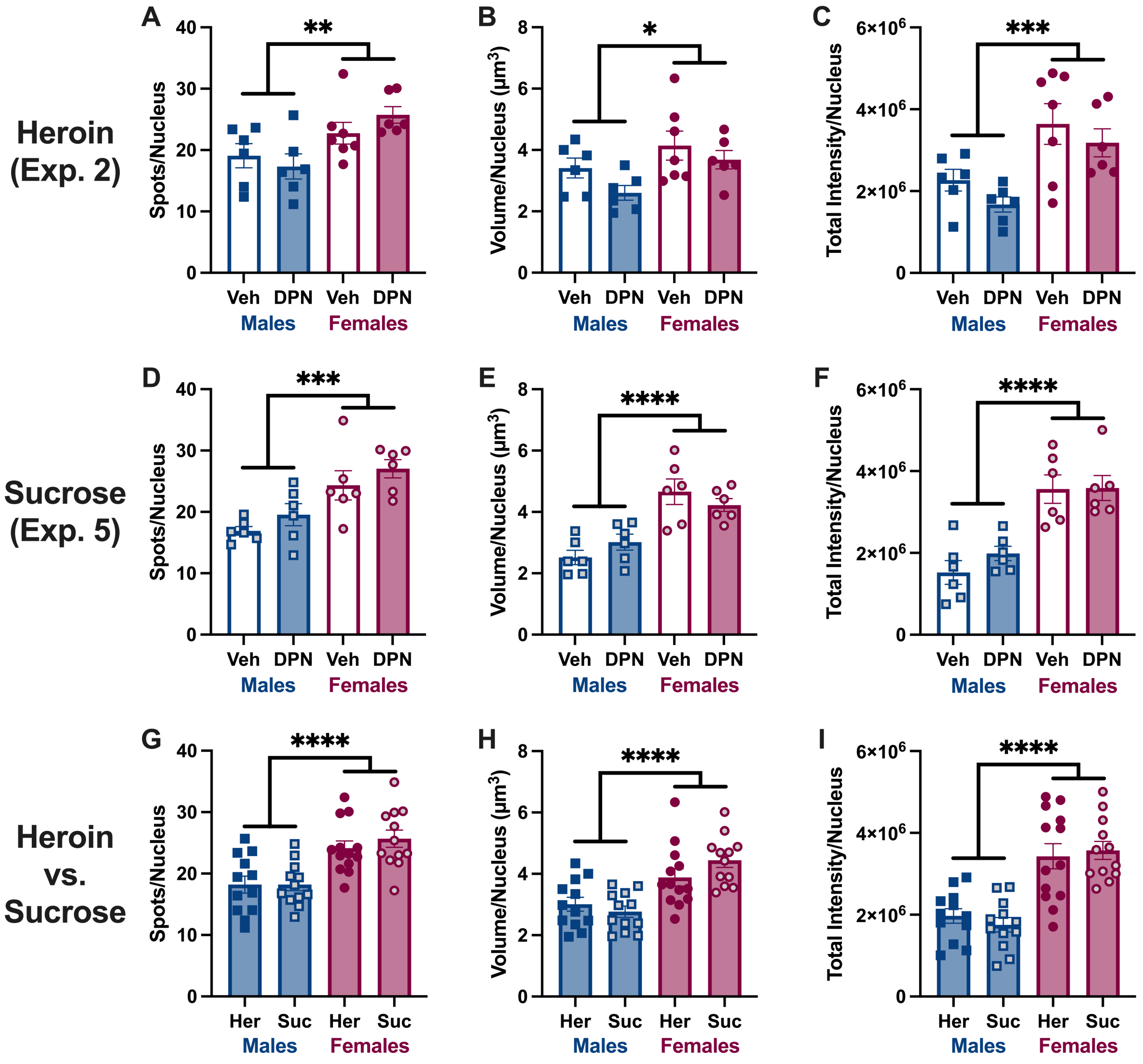

Figure S5. ERβ expression in the basal portion of the basolateral amygdala is not impacted by DPN administration or by heroin or sucrose self-administration. A-C. ERβ spots, spot volume, and total intensity of voxels within spots adjusted for number of nuclei following heroin self-administration. DPN administration did not impact any measure, however there were MEs of sex. D-F. ERβ spots, spot volume, and total intensity of voxels within spots adjusted for number of nuclei following sucrose self-administration. DPN administration did not impact any measure, however there were MEs of sex. G-I. ERβ spots, spot volume, and total intensity of voxels within spots adjusted for number of nuclei following heroin or sucrose self-administration (data collapsed by sex and reproduced from A-F above). Measures did not differ between heroin or sucrose self-administering groups, however there were MEs of sex. All data are shown as mean ±SEM with individual averages for each subject shown. *$p < .05$, **$p < .01$, ***$p < .001$, ****$p < .0001$. BLA, basolateral amygdala; BA, basal portion of the BLA; DPN, diarylpropionitrile (ERβ agonist); ME, main effect.

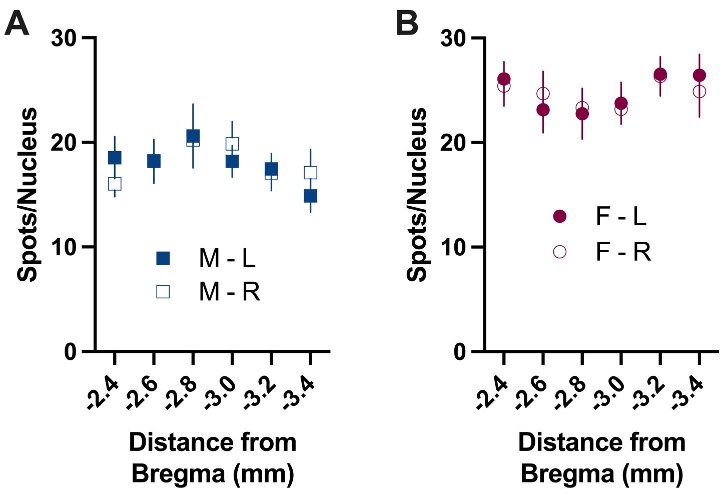

Figure S6. ERβ expression in the basal portion of the basolateral amygdala does not differ across hemispheres. A. ERβ spots, adjusted for number of nuclei, in the left and right BA of males. There were no differences between hemispheres. B. ERβ spots, adjusted for number of nuclei, in the left and right BA of females. There were no differences between hemispheres. Data shown as mean ±SEM. BLA, basolateral amygdala; BA, basal portion of the BLA; L, left hemisphere; R, right hemisphere.

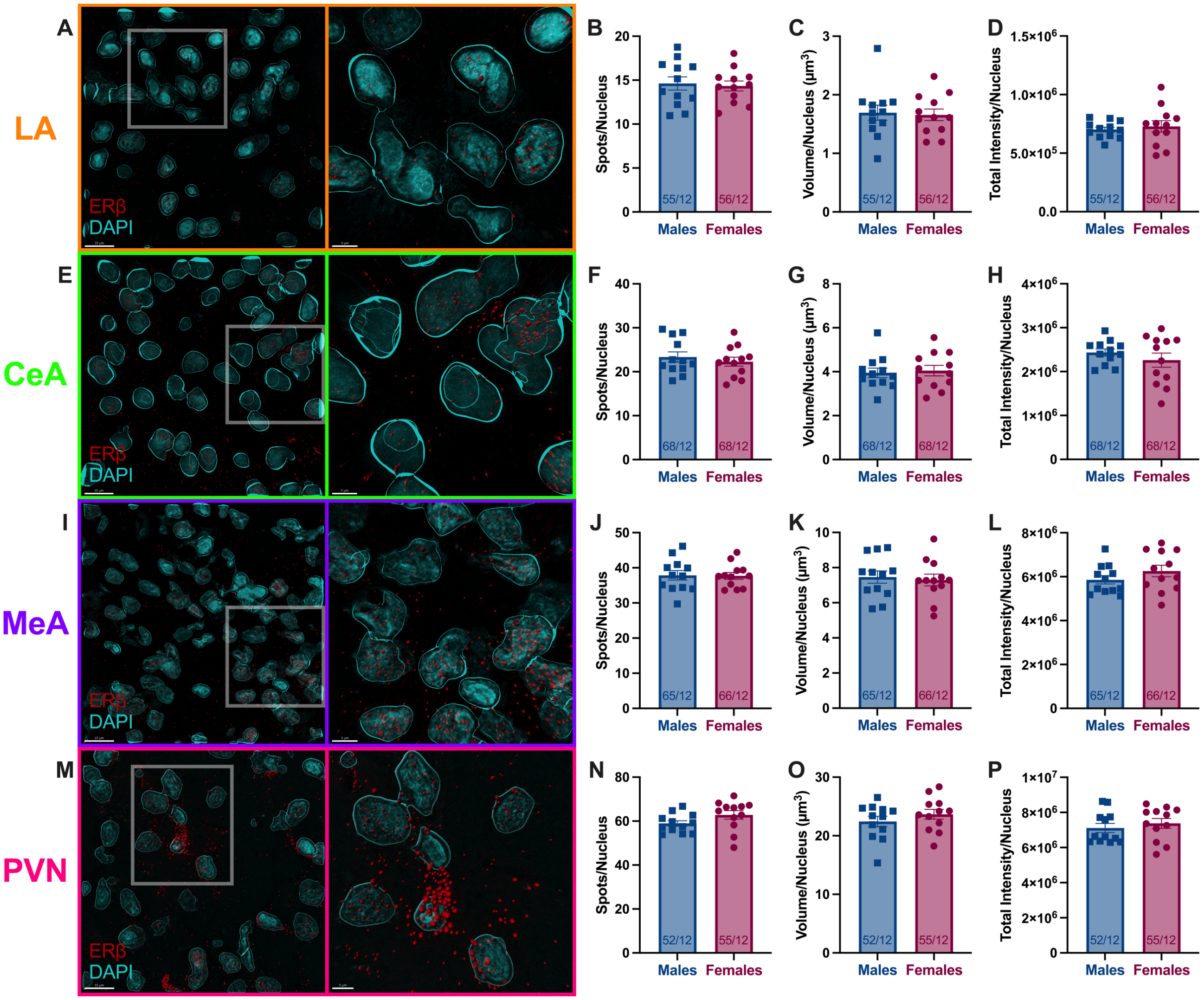

Figure S7. ERβ expression in the LA, CeA, MeA, and PVN is not impacted by sex. A., E., I., M. Representative confocal photomicrographs from the LA, CeA, MeA, and PVN, respectively. Lower magnification Imaris-processed images are shown on the left (scale bars = 15μm). The gray box depicts the higher magnification Imaris-processed images shown on the right (scale bars = 5μm). B., F., J., N. ERβ spots within each region adjusted for number of nuclei. ERβ spots did not differ between males and females in any of these regions. C., G., K., O. ERβ spot volume in each region adjusted for number of nuclei. ERβ spot volume did not differ between males and females in any of these regions. D., H., L., P. Total intensity of voxels within ERβ spots in each region adjusted for number of nuclei. ERβ spot intensity did not differ between males and females in any of these regions. Data shown as mean ±SEM with individual averages for each subject shown. Image and subject *n*'s are shown within columns as Images / Subjects. BLA, basolateral amygdala; CeA, central amygdala; LA, lateral portion of the BLA; MeA, medial amygdala; PVN, paraventricular nucleus of the hypothalamus.
